# Genetic Divergence and Population Structure in Weedy and Cultivated Broomcorn Millets (*Panicum miliaceum* L.) Revealed by Specific-Locus Amplified Fragment Sequencing (SLAF-Seq)

**DOI:** 10.3389/fpls.2021.688444

**Published:** 2021-06-24

**Authors:** Chunxiang Li, Minxuan Liu, Fengjie Sun, Xinyu Zhao, Mingyue He, Tianshu Li, Ping Lu, Yue Xu

**Affiliations:** ^1^School of Life Sciences, Jilin University, Changchun, China; ^2^Key Laboratory for Evolution of Past Life and Environment in Northeast Asia, Ministry of Education, Jilin University, Changchun, China; ^3^Institute of Crop Science, Chinese Academy of Agricultural Sciences (CAAS), Beijing, China; ^4^School of Science and Technology, Georgia Gwinnett College, Lawrenceville, GA, United States

**Keywords:** broomcorn millet, SLAF-seq, domestication, spread route, genetic introgression, Eurasia

## Abstract

Broomcorn millet (*Panicum miliaceum* L.) is one of the earliest domesticated crops in the world. Weedy broomcorn millet [*Panicum ruderale* (Kitag.) Chang or *Panicum miliaceum* subsp. *ruderale* (Kitag.) Tzvel] is thought to be the descendant of the wild ancestor or the feral type of this cereal. The genealogical relationships and genetic divergence among these taxa have not been clarified. In this study, the genetic diversity and population structure of weedy and cultivated broomcorn millets were investigated by using the high-throughput sequencing technology, i.e., the specific-locus amplified fragment sequencing (SLAF-seq). Our analyses consistently revealed both the wild and the feral genotypes in the weedy broomcorn millets. The single nucleotide polymorphisms (SNPs) at the genomic level provided useful evidence to distinguish the wild and the endoferal/exoferal types of weedy broomcorn millets. The genetic divergence revealed between the cultivated broomcorn millet from eastern Eurasia and those from central-western Eurasia was probably derived from either the genetic introgression from weedy broomcorn millets along the spread routes or the founder effect, while the limited gene flow of broomcorn millets from eastern and central-western Eurasia was probably due to the different uses of broomcorn millets and eating habits of the local people.

## Introduction

Broomcorn millet (*Panicum miliaceum* L.) is an important crop broadly cultivated in the semiarid regions of Eurasia and America due to its short growing season and high tolerance of heat and drought. At present, the broomcorn millet has been used as either staple food, bird feed, or forage and is mainly produced in Russia, Ukraine, China, and India. As its companion weed, the weedy broomcorn millet [*Panicum ruderale* (Kitag.) Chang or *Panicum miliaceum* subsp. *ruderale* (Kitag.) Tzvel] is also commonly found in these areas. Both the weedy and cultivated broomcorn millets contain the same chromosome number (2*n* = 4*x* = 36) and can produce fertile offspring by crossing with each other. The morphological characteristics are much similar between weedy and cultivated broomcorn millets with main differences found in pericarp color, plant height, type of panicle, number of branches, seed color, and seed size. One of the most evident morphological variations is the seed color, which is dark yellow in weedy broomcorn millet and yellow or light yellow in cultivated broomcorn millet (Krasavin and Ul’yanova, 1989; Wang, 2006; Zhang et al., 2018b). As the only weedy species in the genus *Panicum* exhibiting a widespread distribution across a region spanning from northern China to western Eurasia, weedy broomcorn millet was thought to be either the descendant of the wild ancestor of cultivated broomcorn millet which had never been domesticated (De Wet, 1992; Wang, 2006) or a feral type formed by either reverse mutations of cultivars (Scholz, 1983) or introgression between cultivars and their wild relatives (You, 1984). It has been suggested that the weedy broomcorn millet in China may be composed of both wild and feral types based on microsatellite markers, while the wild type may harbor the ancestral variations that gave rise to the cultivated broomcorn millet (Xu et al., 2019). However, the feral individuals could not be distinguished from the wild individuals. Further exploration of the morphological and genetic variations between wild and feral types of broomcorn millet are necessary in order to uncover the feral and wild types in weedy broomcorn millet.

As one of the earliest domesticated crops of the world, broomcorn millet has been well documented as domesticated first in northern China at least 8,000 years ago based on a series of archeological evidence (Zhao, 2004; Gansu Provincial Institute of Cultural Relics and Archaeology (GPICRA), 2006; Lu et al., 2009). However, the explicit domestication area of broomcorn millet in northern China is still controversial. On one hand, Loess Plateau was thought to be one of its domestication areas based on archeological discovery in Dadiwan site (about 7,800–7,350 years ago) in Loess Plateau (Gansu Provincial Institute of Cultural Relics and Archaeology (GPICRA), 2006) and genetic studies (Hunt et al., 2018). On the other hand, it was suggested that the Western Liao River Basin in Northeast China might be the domestication area due to the discovery of abundant seed remains of broomcorn millet in Xinglonggou site (8,000–7,500 years ago) in this area (Zhao, 2004). Recently, results of our study based on microsatellite markers suggested that two different genetic clusters were probably derived from Loess Plateau and Northeast China, respectively (Xu et al., 2019). However, the existence of alternative domestication areas for some other genetic clusters was not completely ruled out. Furthermore, two spread routes from the domestication areas in northern China to the western end of Eurasia have been proposed based on archeological and genetic evidence. One is the “Oasis Route” starting from northern China to southern Central Asia, West Asia, and Europe through the “Inner Asia mountain corridor zone” and the other is the “Steppe Route” starting from the Northeast China, passing through the Mongolian Plateau and South Siberia, to Europe (Spengler et al., 2014, 2016; Miller et al., 2016; Xu et al., 2019). However, the detailed pathways of these two routes and possibly other routes are not clear. Therefore, the genetic divergence and population structure in weedy and cultivated broomcorn millets distributed throughout Eurasia need to be further explored in order to obtain more details about the domestication areas and spread routes of cultivated broomcorn millets.

In this study, the genetic diversity and population structure of 106 accessions of weedy and cultivated broomcorn millets distributed throughout Eurasia are investigated by using the high-throughput sequencing technology, i.e., the specific-locus amplified fragment sequencing (SLAF-seq), which has been widely used as an efficient large-scale genotyping method to identify single nucleotide polymorphisms (SNPs) at low cost due to its reduced representation of deep sequencing without sacrificing the genotyping accuracy (Sun et al., 2013). To our knowledge, this is the first comparative study focusing on the genome-wide variations between weedy and cultivated broomcorn millets. The objectives of the present study are (1) to clarify the genealogical relationships between weedy and cultivated broomcorn millets (2) distinguish the wild and feral types in weedy broomcorn millets, and (3) to further explore the domestication and spread routes of broomcorn millets.

## Materials and Methods

### Plant Materials and DNA Extraction

A total of 106 accessions of (9 weedy and 97 cultivated) broomcorn millets were examined in the present study (Supplementary Table 1), provided by the Institute of Crop Science, Chinese Academy of Agricultural Sciences (ICSCAAS). All 9 accessions of the weedy broomcorn millets were collected from China and most of the accessions of the cultivated broomcorn millet were landraces collected from wide geographical locations in Eurasia (Supplementary Table 1). Young leaves of each accession were collected and stored in sterile tubes kept at –80° C. Total genomic DNA was extracted using CTAB method (Paterson et al., 1993). DNA concentration was assessed with a NanoDrop-2,000 spectrophotometer (NanoDrop Technologies Inc., Wilmington, DE, United States). DNA samples were diluted to 50 ng⋅μL^–1^ for high-throughput sequencing.

### Specific-Locus Amplified Fragment Sequencing

The genomic DNA of broomcorn millet was analyzed with specific-locus amplified fragment sequencing (SLAF-seq) (Sun et al., 2013). SLAF-seq was one of the reduced-representation genome sequencing (RRGS) technologies. Compared with the other RRGS technology, i.e., the restriction-site associated DNA sequencing (RAD-seq), SLAF-seq applies a unique pre-designed reduced representation scheme to optimize the efficiency of molecular markers and a double barcode system for large populations (Sun et al., 2013). To optimize the SLAF-seq yields and efficiency, the restriction enzyme combinations were selected *in silico* based on the broomcorn millet (*Panicum miliaceum* L.) reference genome (854.79 Mb)^1^. Two restriction enzymes (*Rsa*I and *Hae*III) (NEB, Ipswich, MA, United States) were selected to digest purified genomic DNA. The genome of *Oryza sativa*^2^ was selected as the control genome to test the accuracy of the restriction enzyme digestion protocol. DNA fragments of 364–414 bp were selected as SLAFs followed by fragment end reparation, dual-index paired-end adapter ligation, PCR amplification, and target fragment selection for SLAF library construction. The paired-end sequencing was performed on the Illumina HiSeqTM 2,500 sequencing platform (Illumina, Inc.; San Diego, CA, United States) at the Biomarker Technologies Corporation in Beijing, China.

### SNP Calling

After the high-throughput sequencing, the raw reads (126 bp) containing either adaptor/primer contamination or low-quality bases were filtered using Seqtk^3^. The high-quality paired-end reads obtained were mapped onto the reference genome (*Panicum miliaceum* L.; see text footnote 1) using the Burrows-Wheeler Aligner (BWA) 0.7.10 with default parameters (Li and Durbin, 2009). SAMtools 1.5 (Li et al., 2009) was used to convert SAM files into BAM and indexed BAM files, and then the Genome Analysis Toolkit (GATK) 3.8 (McKenna et al., 2010) was used to call high-quality SNPs with the option “emitRefConfidence” set to “GVCF” (i.e., true). The low quality SNPs were removed based on the following criteria: QUAL < 30.0; QD < 2.0; FS > 60.0; MQ < 40.0; and SOR > 3.0. Ultimately, consistent SNPs were selected with missing rate at marker level set at ≤ 50% and minor allele frequency (MAF) above 0.05 for further population structure, genealogical, and multivariate analyses to infer genetic diversity and identify selective sweeps.

### Population Structure Analysis and Genealogical Tree Reconstruction

Population structure was investigated using ADMIXTURE_linux-1.3.0 (Alexander et al., 2009). The number of genetic clusters (K) was set from 1 to 10. The ADMIXTURE analysis provided maximum likelihood estimates of the proportion of each sample that was derived from each of the K populations. An accession was attributed to a specific group if this accession had an admixture coefficient ≥ 80% for the group. Genetic distances among the 106 accessions were calculated using the *p*-distance method (Jin and Nei, 1990). Genealogical trees of 106 accessions of broomcorn millets were constructed based on both maximum likelihood (ML) (Stamatakis, 2014) using RAxML 8 (GTR model describing the rate of nucleotide change, nst = 6; gamma distributed rate variation among sites) and neighbor-joining (NJ) based on genetic distance in MEGA X (Sudhir et al., 2016) with bootstrap analysis of 1,000 replicates. The application of these methods of genealogical tree construction was based on previous studies (Li et al., 2017; Su et al., 2017; Zeng et al., 2017; Zhou et al., 2017; Zhang et al., 2017; Yang et al., 2018). Principal component analysis (PCA) based on SNPs was performed with EIGENSOFT software (Price et al., 2006).

### Genetic Diversity and Differentiation Estimates

Genetic diversity indices, including the observed heterozygosity (*H*_O_) and expected heterozygosity (*H*_E_) for weedy and cultivated broomcorn millets were calculated using VCFtools software (Danecek et al., 2011). The analysis of nucleotide diversity (π) was performed using pixy 1.0.0 based on VCF data including invariant sites to avoid biased estimation (Korunes and Samuk, 2021). A VCF file including invariant sites was generated in GATK 3.8 by using the “-allSites” flag in GenotypeGVCFs, with the filtering criteria set to “DP > = 5, GQ > = 40| RGQ > = 40” for invariant sites. The estimates of nucleotide diversity were compared across populations using ANOVA and *post hoc* tests (i.e., LSD). To estimate the linkage disequilibrium (LD) in weedy and cultivated broomcorn millets, the coefficient of linkage disequilibrium (r^2^) of seven weedy accessions and seven cultivated accessions randomly selected among a total of 96 cultivated samples was calculated using PopLDdecay 3.40 (Zhang et al., 2018a) with the distance that the LD decays to half of its maximum value calculated as well. Two weedy samples (i.e., WSX25 and WSX26 from Shanxi, China) identified as feral types and one cultivated sample (i.e., POL2 from Poland) revealed as derived from introgression between weedy and cultivated broomcorn millets were excluded from the LD analysis in order to avoid the interference of these samples. The experimental strategy was based on a previous study in order to avoid the biased results caused by uneven number of samples of weedy and cultivated populations (Zhang et al., 2020). The LD decay graphs of both weedy and cultivated broomcorn millets were plotted. The nucleotide diversity (π) and the pairwise fixation index, i.e., F-statistics (*F*_ST_), across different groups (excluding the “mosaics”) were calculated using pixy (Korunes and Samuk, 2021).

### Putative Selective Sweeps

In order to detect the artificial selection signals between weedy and cultivated broomcorn millets, we scanned the genome in 100-kb sliding windows with a step size of 10-kb to identify the genomic regions with significantly low levels of polymorphisms in the cultivated broomcorn millet based on the fixation index (F-statistics, *F*_*ST*_) and nucleotide diversity ratios (π ratio, π_*w*_/π_*c*_) of weedy and cultivated broomcorn millets (Lam et al., 2010). Highly differentiated genomic regions with the 5% right tails of the *F*_*ST*_ distribution and the top 5% of π ratio were defined as potential selective sweeps (Li et al., 2013). The genes located in these selective sweep regions were considered as candidate genes, which were annotated by the non-redundant (nr) database^4^ at the National Center for Biotechnology Information (NCBI)^5^ and the Swiss-Prot protein database^6^ using BLASTX program (Altschul et al., 1990). The functions of the genes annotated based on the nr database were further classified by using Blat2GO (Conesa et al., 2005) based on the Gene Ontology (GO) database^7^ (Ashburner et al., 2000) and using BlastKOALA (Kanehisa et al., 2016) based on the Kyoto Encyclopedia of Genes and Genomes (KEGG) database (Minoru et al., 2004).

## Results

### Characterization of SLAF Sequencing Data and SNPs

**FIGURE 1:**
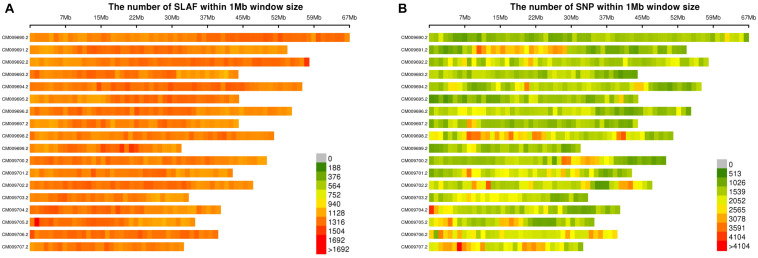
Distribution of SLAF-tags **(A)** and SNPs **(B)** on 18 reference chromosomes of broomcorn millet.

The raw reads were filtered to remove adaptor sequences, empty reads, and low-quality sequences. A total of 326.36 M high-quality clean reads were obtained with a Q30 ratio of 93.48% and a GC content of 45.04%. Approximately 1,096,614 SLAF-tags well-distributed across all 18 chromosomes were identified based on sequence alignment with the reference genome of broomcorn millet (see text footnote 1). The distribution of the SLAF-tags on 18 chromosomes of broomcorn millet was shown in Figure 1A. A total of 483,135 SLAF-tags (∼44.1%) showed polymorphism. The coverage of SLAF-tags for each accession of broomcorn millet ranged from 9.15- to 28.88-fold, with an average of 16.43-fold. A total of 1,523,344 high-quality SNPs obtained were roughly evenly distributed across all 18 chromosomes of broomcorn millet (Figure 1B). The total number of SNPs among the 106 samples of broomcorn millet ranged from 452,349 to 693,152 with the missing rate ranging from 54.50 to 70.31% at individual level. These missing rates are comparable with those reported previously based on SLAF-seq (Chao et al., 2017). The higher individual level missingness may be caused by the enzyme digestion efficiency (91.06%) and the high threshold of marker level missing rate. Furthermore, studies have shown that accurate inference of phylogenies from data matrices containing vast amounts of missing data suggests that it is not missing data but the phylogenetically misleading data and lack of informative data that would cause more inaccurate phylogenies (Rubin et al., 2012). In our study, none of the 106 samples showed abnormally high or low missingness of SNPs, suggesting the unlikely misestimate of the genetic differences of these samples.

### Population Structure of Weedy and Cultivated Broomcorn Millets

**FIGURE 2:**
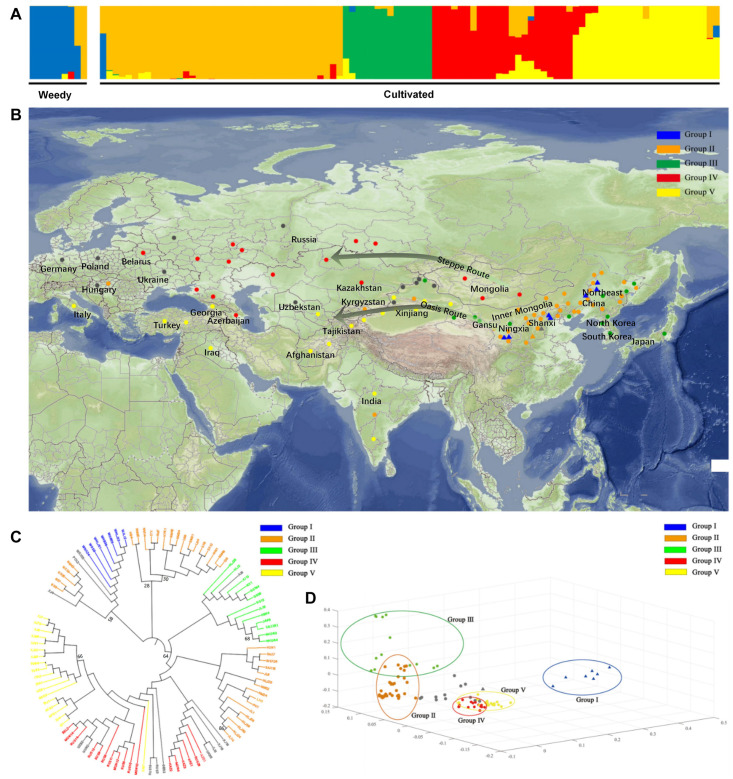
Global distribution and population structure of the weedy and cultivated broomcorn millets. **(A)** Population structure analysis based on 106 accessions of weedy and cultivated broomcorn millets using ADMIXTURE with the optimal clustering number set at *K* = 5. Each accession is indicated by a vertical column and the colored portion (i.e., blue, orange, green, red, and yellow) in each column represents the proportion contributed from ancestral populations. **(B)** Geographic display and summary of ADMIXTURE analysis with the optimal clustering number set at *K* = 5 for 106 accessions of weedy and cultivated broomcorn millets throughout Eurasia. The cultivated accessions from India, Mongolia, and a few accessions from Russia could not be accurately located due to the lack of distribution information within these large areas and are therefore randomly placed inside their distribution areas. Each weedy accession and cultivated accession are represented by a filled triangle and a circle, respectively. Accessions with an admixture coefficient ≥ 80% are attributed to one of the Groups I to V based on ADMIXTURE analysis with each accession labeled with the color of group to which it belongs, while accessions with an admixture coefficient less than 80% are deemed as “mosaics” and colored with gray. **(C)**. The genealogical tree based on neighbor-joining (NJ) of the 106 accessions of weedy and cultivated broomcorn millets. The color of each accession is indicated by the group it belongs to with the “mosaics” marked as gray. The name of each accession starts with W to indicate a weedy accession, while others represent cultivated accessions. Most branches are supported by bootstrap values higher than 90 with six bootstrap values lower than 90 labeled next to the branches. **(D)**. Principal component analysis (PCA) of 106 accessions of weedy and cultivated broomcorn millets. Each weedy accession and cultivated accession are represented by a filled triangle and a circle, respectively. The color of each accession is indicated by the group it belongs to. The “mosaics” are marked as gray. The accessions belong to Group I to V are circled separately.

A total of 126,822 highly consistent SNPs were identified based on further criteria, i.e., minor allele frequency (MAF) ≥ 0.05 and missing rate ≤ 0.50, to examine the population structure of weedy and cultivated broomcorn millets. The population clustering using admixture proportions from 1 to 10 were performed by the ADMIXTURE program. The result of population clustering based on the optimal clustering number of *K* = 5, suggested based on the lowest error rate, was shown in Figure 2A and the admixture coefficients for each accession at *K* = 5 was listed in Supplementary Table 1. The results of *K* = 3, 4, 6, and 7 showing lower error rates were provided in Supplementary Figure 1. The 89 accessions with an admixture coefficient ≥ 80% were attributed to five distinctive groups (i.e., Groups I to V), respectively, and the other 17 accessions each with an admixture coefficient < 80% were deemed as “mosaics.” All accessions in Group I were weedy broomcorn millets, whereas Groups II to V were almost all composed of cultivated broomcorn millets except for one weedy accession (WSX26 from Shanxi, China) assigned to Group II. Furthermore, a weedy accession (WSX25 from Shanxi, China) and a cultivated accession (POL2 from Poland) were revealed to be mixed with large proportion of gene pools in Groups I and II, respectively, probably due to the introgression between weedy and cultivated broomcorn millets. To avoid their interference, these three accessions (i.e., WSX26, WSX25, and POL2) were removed from the comparative analysis of genetic diversity and divergence between weedy and cultivated broomcorn millets and the selective sweep analysis.

The geographic distribution of the 106 weedy and cultivated accessions and the results of ADMIXTURE analysis at *K* = 5 was shown on Figure 2B. Almost all the weedy accessions were attributed to Group I with more than 80% of the blue gene pool components except for two accessions (i.e., WSX25 and WSX26). Furthermore, cultivated accessions accounted for more than 80% of the orange, green, red, or yellow gene pool components and were attributed to Groups II to V, respectively. The accessions of Groups II and III were mainly from eastern Eurasia. Group II included 33 cultivated accessions widely distributed in northern China, three cultivated accessions outside China (each from India, Hungary, and Kyrgyzstan), and one weedy accession (WSX26) from northern China. The 13 accessions of Group III were cultivars from northern China, South Korea, North Korea, and Japan. Additionally, the accessions of Groups IV and V were distributed throughout the central-western Eurasia, including 17 cultivated accessions of Group IV found in Mongolia, Russia, Kazakhstan, and 19 cultivated accessions of Group V broadly distributed in South Asia, Central Asia, West Asia, and Europe, with the distribution of eight accessions in this group from China limited to Xinjiang region. Groups IV and V showed evidently a characteristic distribution of north-south orientation, with Group IV in the north and Group V in the south. Furthermore, some cultivated accessions were shown to be the mixture (i.e., “mosaics”) of gene pools among Groups II to V, indicating the existence of gene flow among these groups.

The genealogical trees were constructed to further explore the genetic relationships among the 106 accessions of weedy and cultivated broomcorn millets attributed to Groups I to V (Figure 2C). The genealogical tree based on ML revealing largely congruent topology as that of the genealogical tree based on NJ was provided in Supplementary Figure 2 with seven cultivated samples in Group III distributed in China allied within Group II mainly distributed in China. The topologies revealed by the genealogical trees based on both the NJ and ML were largely consistent with the results derived from the clustering analysis. All the weedy accessions attributed to Group I were clustered together, showing a close genetic relationship with five cultivated accessions of Group II, i.e., NM43 (Horqin Left Wing Middle Banner, Inner Mongolia), NX19 (Haiyuan, Ningxia), GS35 (Pingliang, Gansu), GS7 (Yuzhong, Gansu), and GS8 (Gulang, Gansu), while one weedy accession (WSX25) and two cultivated accessions (POL2 and XJ4) were between Groups I and II on the genealogical tree. Notably, only one weedy accession WSX26 was attributed to Group II clustered with 15 cultivated accessions, while four accessions (JAP3, SKOR1, NKOR3, and NKOR4) from Japan, South Korea and North Korea showed the close genetic relationship among the cultivated accessions in Group III. The only Indian accession of Group II (ID8) showed close genetic relationship with two accessions (NM1 and NM48) from Inner Mongolia as well as two accessions (SX8 and SX12) from Shanxi, China, while two Indian accessions of Group V (ID1 and ID4) showed close genetic relationship with one accession (TAJ1) from Tajikistan and two accessions (AFG1 and AFG2) from Afghanistan in Central Asia. Furthermore, some cultivated accessions mixed with gene pools among Groups II to V were found mostly in Xinjiang, China or in Europe, where there were a few accessions of Groups II or III, indicating the existence of gene flow among these groups.

Principal component analysis (PCA) was performed on these 106 weedy and cultivated broomcorn millet accessions using EIGENSOFT software (Figure 2D). The three dimensions of principal components (i.e., PC1, PC2, and PC3) explained 14.65, 8.22, and 5.75% of the genetic variation, respectively. Overall, the results of PCA were largely consistent with those derived from both the ADMIXTURE and genealogical analyses, while the accessions of five different groups were fairly separated from each other. On PC1, the weedy and cultivated broomcorn millets were clearly distinguished with only one weedy accession (WSX26) represented with orange triangle clustered with the cultivated accessions and one weedy accession (WSX25) represented with gray triangle located between the weedy and cultivated accessions. This result was in accordance with that derived from the ADMIXTURE analysis showing that WSX25 shared ancestry with the Group I (weedy) and Group II (cultivated) gene pools. On PC2, accessions of Groups II and III were separated from those of Groups IV and V, with the former representing the main genetic diversity of cultivated broomcorn millet in the eastern Eurasia and the latter widely distributed in the central-western Eurasia, indicating that PC2 was related to the geographical distribution of cultivated broomcorn millets. On PC3, accessions of Groups II and III were further distinguished, while no clear distinction was revealed between Groups IV and V, indicating the relatively close genetic distance between cultivated broomcorn millet distributed in north and south parts of central-western Eurasia.

### Genetic Diversity and Divergence Estimation

**FIGURE 3:**
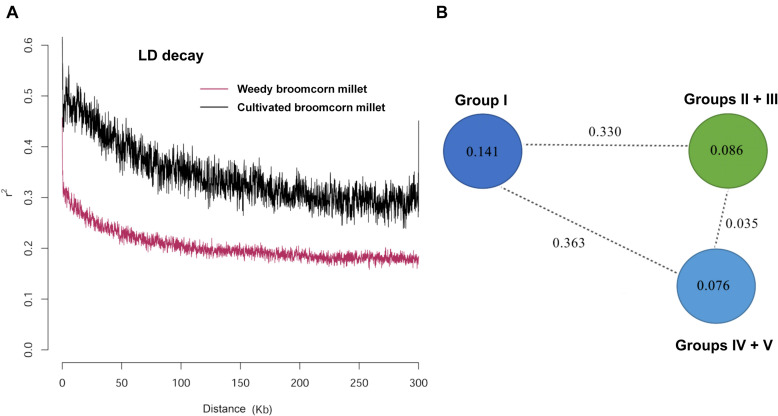
Linkage disequilibrium (LD) in weedy and cultivated broomcorn millets and nucleotide diversity and divergence in Groups I to V identified by ADMIXTURE analysis of 106 accessions of broomcorn millets. **(A)** LD decay estimated by the coefficient of linkage disequilibrium (r^2^) against the distance between polymorphic sites of weedy and cultivated broomcorn millet. **(B)** Nucleotide diversity (π) and fixation index (*F*_ST_) across Group I (including 7 weedy samples collected in China), Groups II + III (containing 52 cultivated samples mainly distributed in eastern Europe), and Groups IV + V (with 37 cultivated samples mainly distributed in central-western Europe). The π measurements of these groups are given in the circles and the value on each dashed line indicates the *F*_ST_ value (*p* < 0.001) between each pair of two groups at the ends of the line.

The genetic diversity parameters including observed heterozygosity (*H*_O_), expected heterozygosity (*H*_E_), and nucleotide diversity (π) for weedy and cultivated broomcorn millets were listed in Table 1. The *H*_O_, *H*_E_, and π of weedy broomcorn millets were all significantly higher than those of cultivated broomcorn millets (*p* < 0.05), indicating that the cultivated broomcorn millet may have experienced significant reduction in genetic diversity during domestication due to the bottleneck effect. The linkage disequilibrium (LD) was estimated and compared between weedy and cultivated broomcorn millets. The coefficient of linkage disequilibrium (r^2^) decayed to half of its maximum value within 60 and 100 kb in weedy and cultivated broomcorn millets, respectively (Figure 3A).

**TABLE 1:** Genetic diversity parameters of weedy and cultivated broomcorn millets.

Accession	N	*H*_O_	*H*_E_	π
Cultivated broomcorn millet	96	0.0131	0.124	0.0954
Weedy broomcorn millet	7	0.0175	0.133	0.1406

*N is the number of samples analyzed, H_O_ is the observed heterozygosity, H_E_ is the expected heterozygosity, and π is the nucleotide diversity.*

In order to investigate the genetic relationship and divergence between weedy and cultivated broomcorn millets and between the cultivated broomcorn millets from eastern and central-western Eurasia, we calculated the nucleotide diversity (π) and the pairwise fixation index (*F*_ST_) across Group I, Groups II + III, and Groups IV + V (Figure 3B). This grouping strategy was based on the results of ADMIXTURE analysis (Figure 2A and Supplementary Table 1). Specifically, most of accessions of broomcorn millet were categorized into Groups I–V, except for some “mosaics” and almost all the weedy broomcorn millets were attributed to Group I, while the cultivated broomcorn millets from eastern Eurasia clustered into Groups II and III and most cultivated broomcorn millets from central-western Eurasia clustered into Groups IV and V. The results showed that the nucleotide diversity of Group I (0.141) was higher than 0.086 and 0.076 of Groups II + III and Groups IV + V, respectively. Furthermore, larger genetic divergence was observed between Group I and Groups II + III (*F*_ST_ = 0.330) and between Group I and Groups IV + V (*F*_ST_ = 0.363) than that between Groups II + III and Groups IV + V (*F*_ST_ = 0.035), indicating a relatively closer relationship between cultivated broomcorn millets from eastern and central-western Eurasia than that between weedy and cultivated broomcorn millets.

### Identification of Artificial Selection Signals in Domestication

**FIGURE 4:**
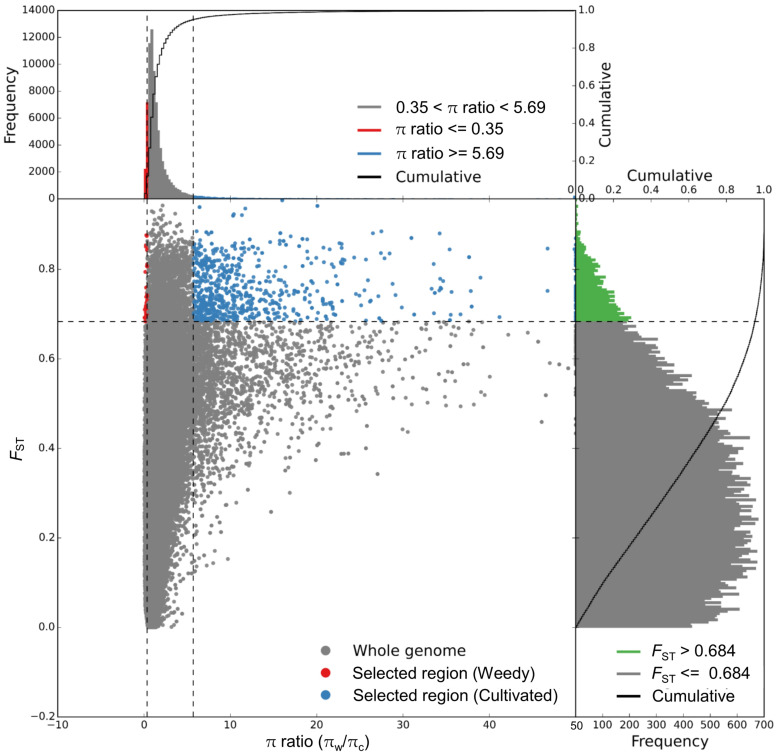
Genomic regions with selective sweep signals in cultivated broomcorn millet genome showing distribution of π ratio (π_w_/π_c_) and *F*_ST_ values of 100-kb windows with 10-kb steps. Blue dots represent windows fulfilling the requirement (i.e., *F*_ST_ ≥ 0.684 and π ratio ≥ 5.69) of selected regions in genome of cultivated broomcorn millet.

The genomic regions with both π ratio and *F*_ST_ values in the top 5% were considered as having been subjected to selective sweeps (Figure 4). A total of 933 putative selective sweeps were identified including 2,486 genes in the cultivated broomcorn millet genome. These results suggested that the selective sweeps caused by artificial selection or natural selection existed during the domestication of broomcorn millet.

**FIGURE 5:**
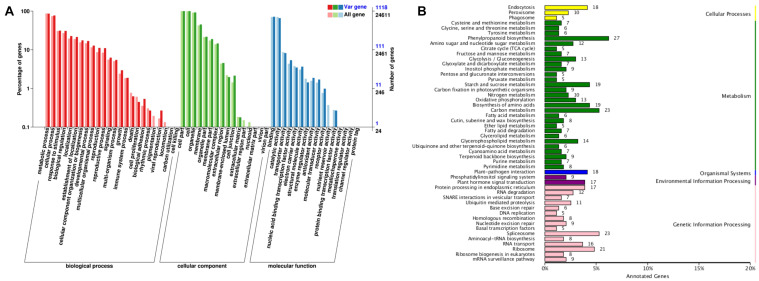
Functional annotations of 2,486 genes identified in selective sweep regions of cultivated broomcorn millet genome. **(A)** Annotation based on GO database. The left *Y*-axis indicates the proportion of genes in the three main categories of GO classification and the right *Y*-axis indicates the number of genes in each category. “All gene” represent all genes in the GO database and “Var gene” represent the selected genes annotated to the GO database. **(B)** Annotation based on KEGG database. The *Y*-axis indicates the top 50 metabolic pathways, the *X*-axis indicates the numbers of genes annotated to the pathways and its proportion to the total number of genes annotated.

The 2,486 genes identified in selective sweeps were annotated to a total of 42 Gene Ontology (GO) terms with 20 (47.62%), 11 (26.19%), and 11 (26.19%) revealed in three GO categories, i.e., biological processes, cellular component, and molecular function, respectively (Figure 5A). Genes involved in metabolic processes (961) and cellular processes (861) were the top two most abundant subcategories in the biological process, while the cell part (1,118), cell (1,117), and organelle (1,036) contained the most highly represented genes in the cellular component category. The binding (799) and catalytic activity (735) represented the majority of genes in the category of molecular function.

The 2,486 genes identified in the selective sweeps were further enriched in the metabolic pathways based on the Kyoto Encyclopedia of Genes and Genomes (KEGG) database (Figure 5B). A total of 434 genes were annotated in 104 biological pathways in the KEGG database. The largest number of genes were annotated into the pathways of phenylpropanoid biosynthesis (27; 6.2%), followed by carbon metabolism (23; 5.3%), spliceosome (23; 5.3%), and ribosome (21; 4.8%). Among the five groups of pathways in the KEGG database, the metabolism contained the highest number of genes (234; 53.9%) and the organismal systems contained the least number of genes (21; 4.8%).

Results of Swiss-Prot analysis showed that a total of 21 genes were annotated to genes involved in domestication of cereal crops, including (1) *rna17169* (in chromosome CM009694.2) annotated to the *DAR1* gene controlling seed and organ size by restricting the period of cell proliferation in *Arabidopsis thaliana* (Li et al., 2011) and *Oryza sativa* (Mumtaz, 2017); (2) *rna22663* (in CM009695.2) annotated to *BLH9* (encoding BEL1-like homeodomain), affecting the development of apical meristem, stem, embryo seat, and inflorescence development in *Arabidopsis thaliana* (Smith and Hake, 2003; Cole et al., 2006) and *Zea mays* (Cao et al., 2015), and (3) a total of 19 gene regions (i.e., *rna8454*, *rna8509*, *rna8510*, *rna8511*, *rna8512*, and *rna9620* in CM009691.2, *rna12076* and *rna9941* in CM009692.2, *rna18186* in CM009694.2, *rna21536* in CM009695.2, *rna26025* in CM009696.2, *rna32291* in CM009698.2, *rna34423*, *rna34423*, and *rna34496* in CM009699.2, *rna41406* in CM009701.2, *rna44694*, *rna44695*, and *rna44696* in CM009702.2, and *rna46779* in CM009703.2) annotated to *AP2/ERF* gene family, which were involved in diverse types of biological functions, including the increases of seed weight, total seed protein, and total seed oil contents as well as the floral organ development and flowering time in *Arabidopsis thaliana* (Jofuku et al., 2005; Yant et al., 2010), disease resistance (Chen and Guo, 2008) and response to abiotic stresses (Cheng et al., 2013).

## Discussion

### Genetic Relationship Among Wild, Feral, and Cultivated Types of Broomcorn Millets

In the present study, the genome-wide diversity in the weedy and cultivated broomcorn millet accessions with broad geographic distributions was investigated using SLAF-seq method. The genetic diversity indices (*H*_O_, *H*_E_, and π) of the cultivated broomcorn millets were significantly lower than those of the weedy broomcorn millets across the entire genome (*p* < 0.05) (Table 1). Similarly, higher genetic diversity in weedy broomcorn millet was also observed in our previous comparative studies of weedy and cultivated broomcorn millet based on microsatellite markers (Xu et al., 2019). The decreased genetic diversity in cultivated broomcorn millet compared with that of weedy broomcorn millet is consistent with the hypothesis that domestication process causes a genetic bottleneck, which decreases genetic diversity in crops in comparison to their progenitors (Doebley et al., 2006). Therefore, the significantly higher genetic variation in weedy broomcorn millet revealed in this study provides new evidence to support our previous speculation that weedy broomcorn millets are probably composed of wild types harboring ancestral variations that give rise to the cultivated broomcorn millet, suggesting that the wild genetic resources should be preserved as important resources for improvement of crop characteristics in the future crop breeding.

Results based on the analyses of population structure, genealogical relationship, PCA, and pairwise *F*_ST_ across Groups I, II + III, and IV + V identified by ADMIXTURE analysis have consistently shown that there was evident genetic distinction between weedy and cultivated broomcorn millet populations, probably due to domestication bottleneck and artificial selection (Figures 2, 3B). The LD decay values within the broomcorn millet (60 kb and 100 kb in weedy and cultivated broomcorn millets, respectively) are similar to those of other inbreeding species, including sorghum (∼150 kb) (Morris et al., 2013), rice (∼123 kb for *indica* and 167 kb for *japonica*) (Huang et al., 2012), soybean (∼80 kb in wild soybeans and 130 kb in cultivated soybeans) (Wang et al., 2016), and foxtail millet (∼100 kb) (Jia et al., 2013), which are generally higher than those of out-crossing species, such as maize (∼30 kb) (Hufford et al., 2012) and apple (less than 1 kb) (Ma et al., 2017). Furthermore, the lower LD decay value revealed in weedy broomcorn millet than that in cultivated broomcorn millet, i.e., LD decays more slowly in the cultivated broomcorn millets compared to the weedy broomcorn millets, also indicates the existence of the effect of domestication bottleneck and artificial selection from weedy to cultivated broomcorn millet (Figure 3A).

In order to further detect the signals of artificial selection in the genomic sequences of cultivated broomcorn millet, a total of 933 selective sweeps were identified, including 2,486 candidate genes. Many of these candidate genes were involved in various categories of biological functions (e.g., associated with *DAR1*, *BLH9*, and *AP2/ERF* gene family) based on Swiss-Prot and GO annotations and multiple metabolic pathways based on KEGG enrichment analysis. These results indicate that there was reduction in genetic diversity caused by either artificial selection or natural selection/local adaptation in the domestication process of broomcorn millet. Future investigations are necessary to explore the possibility that some of these 2,486 candidate genes may be related to domestication of broomcorn millet. Further studies are needed to investigate and ultimately identify the genes related to domestication in the regions with reduction in genetic diversity of the broomcorn millet genome. To date, studies have identified genes involved in domestication in cereal crops. For example, *DAR1* was reported to control seed and organ size by regulating the period of cell proliferation in *Arabidopsis thaliana* (Li et al., 2011) and *Oryza sativa* (Mumtaz, 2017). Our results revealed that one gene (*rna17169*) identified in the selective sweeps was annotated to the gene of *DAR1*, suggesting that this gene may be involved in the seed enlargement during domestication of broomcorn millet. Another gene (*rna22663*) in the selective sweeps was annotated to *BLH9* (encoding BEL1-like homeodomain), which was reported to affect the development of apical meristem, stem, and embryo seat in *Arabidopsis thaliana*. Specifically, the inflorescence of a mutant of this gene in *Arabidopsis thaliana* showed abnormal internodes, small, deformed, and clustered pods (Smith and Hake, 2003; Cole et al., 2006). Furthermore, three homologous genes of *BLH9* (*ZmBELL8*, *ZmBELL13*, and *ZmBELL14*) were also found to be involved in the regulation of inflorescence development in *Zea mays* (Cao et al., 2015). These results suggest that gene *rna22663* in broomcorn millet might be a candidate gene associated with the transformation from scattered panicle in weedy broomcorn millet to compact panicle type in cultivated broomcorn millet during domestication. Moreover, a group of 19 gene regions (*rna8454*, *rna8509*, *rna8510*, *rna8511*, *rna8512*, *rna9620*, *rna12076*, *rna9941*, *rna18186*, *rna21536*, *rna26025*, *rna32291*, *rna34423*, *rna34423*, *rna34496*, *rna41406*, *rna44694*, *rna44695*, *rna44696*, and *rna46779*) were annotated to the gene family of *AP2/ERF*, which were associated with a diverse types of biological functions, including the increases of seed weight, total seed protein, and total seed oil contents as well as the floral organ development and flowering time in *Arabidopsis thaliana* (Jofuku et al., 2005; Yant et al., 2010), with some genes involved in disease resistance (Chen and Guo, 2008) and response to abiotic stresses (Cheng et al., 2013). It is reasonable to hypothesize that many of these biological functions played important roles in the domestication of broomcorn millet. Future studies are necessary to explicitly verify the functions of these potential candidate genes of the selective sweeps in the domestication of broomcorn millet.

Generally, the wild populations of many crops are considered as admixture of both wild and feral types with the feral type further divided into endoferal and exoferal types. The endoferal types are formed from a single domesticated lineage and the exoferal types are derived via admixture, either among domesticated lineages or between domesticated taxa and their wild relatives (Barazani et al., 2016; Wang et al., 2017; Gering et al., 2019; Page et al., 2019). Although both the wild and the feral types were found in weedy broomcorn millet in our previous investigation (Xu et al., 2019), the genetic difference between wild and feral types has not been clarified. In the present study, the genetic polymorphism of weedy broomcorn millet was further explored at genomic level. The results showed that the accessions of weedy broomcorn millet were clustered into Group I with two exceptions that WSX26 was assigned to Group II and WSX25 was the admixture with the large proportion of gene pools for Groups I and II (Figure 2A). Similarly, WSX 26 was clustered with the cultivated accessions on both the genealogical tree and PCA plot, indicating its close relationship with the cultivated broomcorn millets, whereas WSX25 was located between the weedy and cultivated accessions, suggesting its origin of hybridization. These results suggest that the accessions of weedy broomcorn millet in Group I probably represent the wild types, while the weedy accession WSX26 might belong to endoferal type escaping from cultivars and WSX25 be an exoferal type formed by the introgression from cultivated broomcorn millets to weedy broomcorn millets implying that the SNPs at the genome level can provide useful evidence to distinguish the wild and the endoferal/exoferal types of weedy broomcorn millets. We note that more weedy accessions of broomcorn millet and rigorous testing of multi-population F-statistics (Peter, 2016) of both weedy and cultivated accessions are required to provide further support for the distinction of genetic types of weedy broomcorn millet.

### Domestication and Subsequent Introgression of Broomcorn Millets

A series of broomcorn millet remains prior to 5,000 BC have been found in northern China. For example, abundant charred grains of broomcorn millet were found at the Dadiwan site (about 7,800–7,350 years ago) in Loess Plateau (Gansu Provincial Institute of Cultural Relics and Archaeology (GPICRA), 2006). Therefore, it is naturally speculated that the cultivated broomcorn millets were likely domesticated in the Loess Plateau. This speculation is supported by several lines of genetic evidence (Hu et al., 2009; Hunt et al., 2018). Furthermore, a large number of carbonized grains of broomcorn millet were found at the Xinglonggou site (8,000–7,500 years ago) in Northeast China, indicating that the Xiliao River Basin in Northeast China may also be a domestication area for cultivated broomcorn millet (Zhao, 2004). Moreover, our recent molecular and genetic investigations suggested that different genetic types of cultivated broomcorn millet may be derived from the Loess Plateau and the Northeast China, respectively (Xu et al., 2019).

In this study, the genetic diversity and structure of 106 accessions of broomcorn millet from East, Central, West, and South Asia, Siberia, and Europe were analyzed at the genomic level. The results of ADMIXTURE analysis with the optimal clustering number set at *K* = 5 (Figures 2A,B) showed that the weedy accessions were almost all classified into Group I, while the cultivated accessions were categorized into Groups II to V. On the genealogical tree based on NJ, all weedy accessions of Group I were clustered together, showing the close genetic relationship with cultivated accessions of Group II from Horqin Left Wing Middle Banner in Inner Mongolia, Haiyuan in Ningxia, Pingliang, Yuzhong, and Gulang in Gansu (Figure 2C). These areas are geographically located in the Northeast China and the Loess Plateau, indicating that some primitive genetic types of cultivated broomcorn millet were preserved locally, supporting the point of view that these two areas may be the domestication areas for cultivated broomcorn millet (Xu et al., 2019).

The accessions of Groups II + III were distributed in the eastern Eurasia, while the accessions of Groups IV + V were distributed in the central-western Eurasia (Figure 2B). Although they were geographically isolated, the genetic divergence between Groups II + III and Groups IV + V were much less than those either between Groups II + III and Group I or between Groups IV + V and Group I (Figure 3B). These results suggested that the genetic divergence between eastern and central-western cultivated broomcorn millet probably developed after the domestication from weedy broomcorn millets. Furthermore, the genealogical tree based on NJ revealed that the cultivated accessions of Group IV and V showed close genetic relationship with 15 accessions of cultivated broomcorn millets of Group II (Figure 2C) with 11 accessions distributed in Northeast China, suggesting that central-western cultivated broomcorn millets may be derived from cultivated broomcorn millet in Northeast China or its surrounding areas. These results are consistent with those reported in our previous studies based on microsatellite markers, suggesting that some cultivated broomcorn millets spreading throughout the Eurasian steppe may have been domesticated in Northeast China (Xu et al., 2019). We note that we could not rule out the possible existence of other areas where the central-western cultivated broomcorn millets were derived from due to the limitation of selected samples, especially the lack of accessions of weedy broomcorn millet outside China in the present study. The genetic divergence between eastern and central-wester cultivated broomcorn millet could be explained with two possible scenarios. One possibility is that the central-western cultivated broomcorn millets were formed through genetic introgression from weedy broomcorn millets to eastern cultivated broomcorn millets in the process of its westward spread. The study on the remains of broomcorn millet found in Xiaohe site in Xinjiang, China, was consistent with this hypothesis, suggesting that the European broomcorn millets were probably derived from the hybridization between Xiaohe type and wild type of this cereal (Li et al., 2016). The other possibility is that the central-western cultivated broomcorn millet may be partly formed by the founder effect of the ancestors of eastern cultivated broomcorn millets, which is supported by a previous study based on genetic diversity of 98 cultivated samples of broomcorn millets throughout Eurasia, proposing that the cultivated broomcorn millets in western Eurasia may be derived from the founder effect of cultivated broomcorn millets originated from China (Hunt et al., 2011). More accessions distributed throughout Eurasia based on multi-population F-statistics (Peter, 2016) are needed to provide further support for these conclusions.

### Spread Routes of Cultivated Broomcorn Millets

After domestication, broomcorn millet spread rapidly throughout the Yellow River Basin during the Yangshao culture period (7000–5000 BP) and became an important food source in the Yellow River Basin. Subsequently, the broomcorn millet spread westward to Hexi Corridor, Xinjiang, and Central Asia at around 5000–4000 BP (Zhao et al., 2013; Spengler et al., 2014; Yang et al., 2014; Zhou et al., 2016, 2020) and dispersed along the “Inner Asian Mountain Corridor” (Miller et al., 2016). Finally, it appeared in Western Asia and Europe at about 3500 BP (Motuzaite-Matuzeviciute et al., 2013). Additionally, there was a secondary phase of westward expansion for cultivated broomcorn millet on the northern Eurasian steppe at the late 2nd and early 1st millennia BC (Hunt et al., 2018). Our previous studies on the genetic diversity of broomcorn millet throughout Eurasia based on microsatellite markers proposed that the westward spread of cultivated broomcorn millet probably reached Europe through either “Steppe Route” or “Oasis Route” (Xu et al., 2019). More details of these routes and possibly some other alternative routes to Northeast Asia and South Asia for this cereal warrant further investigations.

Our results in the present study provided novel evidence to support the westward spread route, which is an essential path for cultivated broomcorn millets to spread from northern China. As shown in Figure 2B, the cultivated broomcorn millets of Groups II and III spread to the large areas of northern China after domestication, becoming the dominant type of this cereal in China. A few accessions of cultivated broomcorn millets entered northwest Xinjiang through Hexi Corridor, including individuals of Group II appeared in the Tianshan region to the north of Tarim Basin and individuals of Group III appeared in the Altay region in northern Xinjiang. The former was also distributed in the Central Asia and Europe, while the latter was not found to the west of Xinjiang, indicating that Group II probably further spread westward along the “Oasis Route” to the north of Tarim Basin and finally reached Europe through Central Asia, while Group III was not spreading outside China. It is noted that the spread of cultivated broomcorn millets along the south edge of Tarim Basin is not clear due to the lack of local samples in this study. Based on archeological evidence, an eastward spread route of wheats was proposed from West Asia to Central Asia, Pamir Plateau, the “Oasis Route” on both sides of Tarim Basin, the Hexi Corridor, and finally reached the Loess Plateau (Zhao, 2015), which is coincidently almost the same as the westward “Oasis Route” of broomcorn millet, suggesting a possible bidirectional exchange of crops on this route.

With our above proposal that the cultivated broomcorn millets of Groups IV and V were probably derived from Northeast China, the wide distributions of accessions of cultivated broomcorn millet in Group IV in the vast area of Eurasian steppe from Mongolian Plateau, Kazakhskiy Melkosopochnik, and West Siberian plain, to the Eastern European Plain and the north of Caspian Sea and Black sea suggest that the cultivated broomcorn millets of Group IV might have spread westward along this “Steppe Route.” Furthermore, the cultivated broomcorn millets of Group V showed vast distribution to the south of distribution areas of Group IV, including the areas from Tianshan region to the north of Tarim Basin in Xinjiang, the southern Central Asia, the Western Asia, and the south region of the Caspian Sea and the Black Sea in Europe. The genealogical analysis showed that cultivated broomcorn millets of Group V were closely related to some individuals of Group IV (Figure 2C), suggesting that these accessions may have shared common ancestry. However, it is still not clear where the Group V cultivars were derived from and how they subsequently spread to the vast areas from Xinjiang, China, to the south region of the Caspian Sea and the Black Sea in Europe. More samples are needed from these local areas in order to further explore the details of these spread routes.

The distributions of cultivated broomcorn millets of Groups II + III and Groups IV + V are limited in eastern and central-western Eurasia, respectively, while the admixture with large proportion of gene pools between Groups II + III and Groups IV + V was not common, indicating that the gene flow between these lines is limited (Figures 2A,B). This may be due to the different uses of broomcorn millets and eating habits of the local people in these areas. For example, the cultivated broomcorn millets in eastern Eurasia (including North and Northeast China, Japan, South Korea, and North Korea) are mainly waxy type, while those in central-western Eurasia (including Northwest China, Central and West Asia, Siberia, and Europe) are mostly non-waxy due to the dietary preference (Wang, 2006). Additionally, in some parts of Europe and North America, the broomcorn millet is mainly used as bird food but not human staple food (Baltensperger, 2002). Furthermore, Groups IV and V are characterized by having a distribution of north-south orientation with Group IV in the north and Group V in the south. This may be related to the environmental and climate variations between high and low latitude areas. The physiological factors of cultivated broomcorn millets in these two areas, such as photoperiod, growth cycle, and stress resistance, are subjected to long-term natural selection, resulting in genetic differentiation between cultivars in northern and southern areas. Similar results have also been obtained in the studies of other crops. For example, the microsatellite analysis of 313 *japonica* rice varieties from 20 countries revealed that the varieties within similar latitudes showed close genetic relationship, while the rice varieties with large difference in latitudes showed increased genetic differentiation (Shu et al., 2009). Similarly, it was reported that the genotypes of soybean also varied with latitude (Lu et al., 2017).

It is suggested that there are two possible sources of cultivated broomcorn millets in India. One is from the further southward disperse of the broomcorn millets spreading westward through Tianshan region (Hunt and Jones, 2008) and the other is probably from northern China through valleys of Hengduan Moutains (Shi, 2000). In the present study, the cultivated broomcorn millets in India are attributed into Groups II and V. Accessions in Group II are the main types of broomcorn millet in China and seldom appear on the westward route of broomcorn millets. The accessions of Group II appearing in India showed close genetic relationship with some accessions in Group II from Inner Mongolia and Shanxi in China (i.e., NM1, NM48, SX8, and SX12) on the genealogical tree (Figure 2C), indicating that some cultivated broomcorn millets in India probably spread directly from northern China. As the main type on the westward spread route, accessions of Group V are also found in India, which are closely related to the Group V accessions in Tajikistan and Afghanistan (TSJ1, AFG1, and AFG2), as shown on the genealogical tree (Figure 2C), suggesting that some of the cultivated broomcorn millets of Group V may further spread southward to South Asia from Tajikistan and Afghanistan on their westward spread route. These results further indicate that the two sources of Indian cultivated broomcorn millet may exist or spread at the same time.

Our results showed that the cultivated broomcorn millets in Japan, South Korea, and North Korea all belong to Group III (Figure 2B) and are closely related (Figures 2B,C) to three accessions (JL18, HB34, and GS12) each from Northeast China, North China, and Hexi corridor, respectively, on the genealogical tree, making it difficult to infer their genetic sources based on the present study (Figure 2C). These results are consistent with those reported previously, revealing that broomcorn millets from Korea and Japan showed close genetic relationship and many landraces from Hokkaido and northern Honshu were genetically similar to those from Northeast China (Hunt et al., 2018). Furthermore, the phylogeny of cultivated broomcorn millets in Japan based on the study of *Waxy* genes showed that Japanese non-waxy broomcorn millets were introduced from the Far East of Russia and Northeast China, while the waxy broomcorn millets in Japan were derived from Korean Peninsula and the Far East of Russia (Araki et al., 2012). Moreover, Shandong and Liaodong Peninsula was also regarded as the probable source of broomcorn millets in Korea and Japan (You, 1984). To date, the genetic sources of cultivated broomcorn millets in Korea and Japan still remain controversial, with three possible candidate regions, including Northeast China, Shandong and Liaodong Peninsula, and Far East of Russia. More studies of broomcorn millet landraces in Northeast Asia are needed in order to explicitly reveal the spread routes in this area.

## Conclusion

In the present study, we investigated the genetic diversity and population structure of a total of 106 accessions of weedy and cultivated broomcorn millets distributed throughout Eurasia using the high-throughput sequencing technology (i.e., SLAF-seq). In this first comparative study focusing on the genome-wide variations between weedy and cultivated broomcorn millets, our results showed that both the wild and the feral types were revealed in the weedy broomcorn millets explored in this study. This study has demonstrated that the SNPs at the genomic level provided useful evidence to distinguish the wild and the endoferal/exoferal types of weedy broomcorn millets. We further suggested that the genetic divergence revealed between the cultivated broomcorn millet from eastern Eurasia and those from central-western Eurasia was probably derived from either the genetic introgression from weedy broomcorn millets along the spread routes or the founder effect. The significantly higher genetic variation in weedy broomcorn millet revealed in this study suggests that the wild genetic resources should be preserved as important resources for improvement of crop characteristics in the future crop breeding.

## Data Availability Statement

The original contributions presented in the study are publicly available. This data can be found here: NCBI with accession numbers of SAMN18616674 to SAMN18616779.

## Author Contributions

CL and ML: conceptualization and investigation. XZ, MH, and TL: methodology. XZ and MH: software. YX, FS, CL, and ML: validation and formal analysis. ML and PL: resources. CL and YX: data curation. CL, YX, FS, ML, XZ, MH, TL, and PL: writing—original draft preparation. CL, YX, and FS: writing—review and editing. XZ: visualization. YX: supervision, project administration, and funding acquisition. All authors have read and agreed to the published version of the manuscript.

## Conflict of Interest

The authors declare that the research was conducted in the absence of any commercial or financial relationships that could be construed as a potential conflict of interest.

## Footnotes

1 https://www.ncbi.nlm.nih.gov/assembly/GCA_003046395.2

2 http://rapdb.dna.affrc.go.jp/

3 https://github.com/lh3/seqtk

4 ftp://ftp.ncbi.nih.gov/blast/db/FASTA/nr.gz

5 https://www.ncbi.nlm.nih.gov/

6 http://www.uniprot.org/downloads

7 http://www.geneontology.org

## Supplementary Material

Supplementary Figure 1 Population structure analysis based on 106 accessions of weedy and cultivated broomcorn millets using ADMIXTURE with the optimal clustering number set at *K* = 3, 4, 6, and 7, respectively. Each accession is indicated by a vertical column and the colored portion (i.e., blue, orange, green, red, and yellow) in each column represents the proportion contributed from ancestral populations.

Supplementary Figure 2 The genealogical tree based on maximum likelihood of the 106 accessions of weedy and cultivated broomcorn millets.

Supplementary Table 1 Detailed information of the 106 accessions of cultivated and weedy broomcorn millets.

Supplementary Appendix Code availability.
