# Supplementary figures and images for: Genetic Divergence and Population Structure in Weedy and Cultivated Broomcorn Millets (Panicum miliaceum L.) Revealed by Specific-Locus Amplified Fragment Sequencing (SLAF-Seq)
Source: Front Plant Sci. 2021 Jun 24;12:688444. doi: 10.3389/fpls.2021.688444 (PMC8264369; doi:10.3389/fpls.2021.688444)

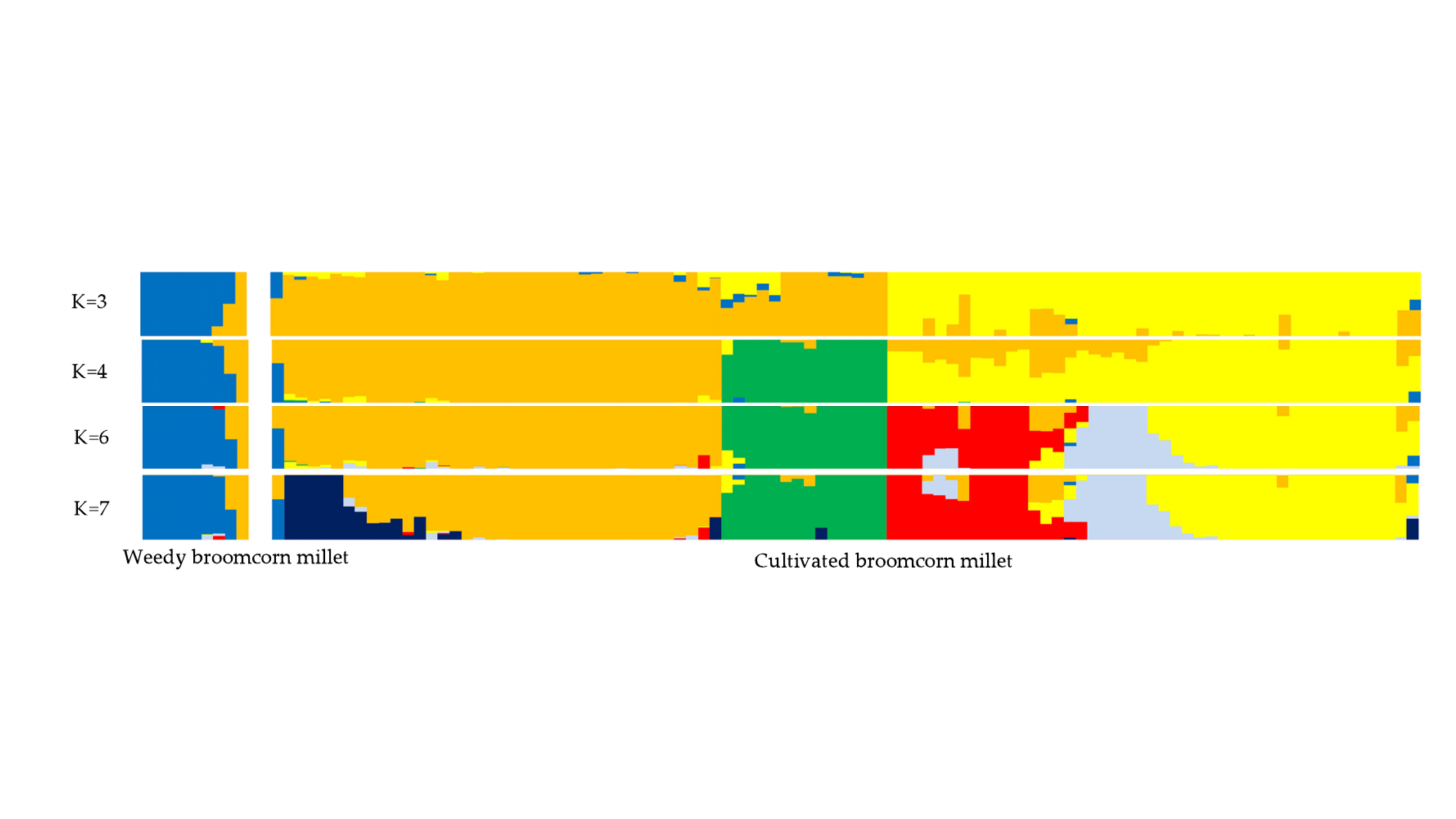

Supplement: Supplementary Figure 1 — Population structure analysis based on 106 accessions of weedy and cultivated broomcorn millets using ADMIXTURE with the optimal clustering number set at K = 3, 4, 6, and 7, respectively. Each accession is indicated by a vertical column and the colored portion (i.e., blue, orange, green, red, and yellow) in each column represents the proportion contributed from ancestral populations. [file Image_1.JPEG]

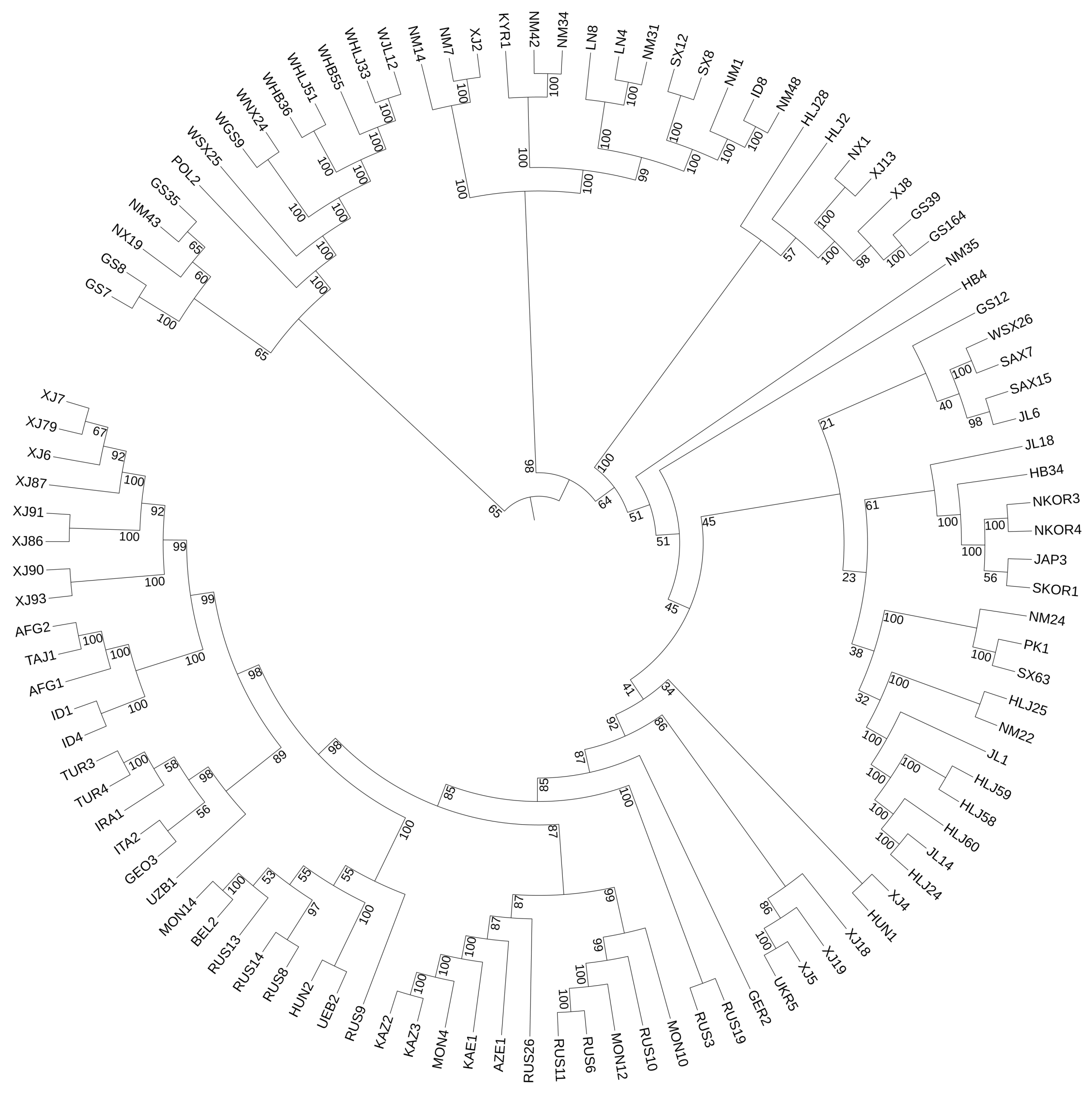

Supplement: Supplementary Figure 2 — The genealogical tree based on maximum likelihood of the 106 accessions of weedy and cultivated broomcorn millets. [file Data_Sheet_1.PDF]
