# Supplementary material for: Genetic Divergence and Population Structure in Weedy and Cultivated Broomcorn Millets (Panicum miliaceum L.) Revealed by Specific-Locus Amplified Fragment Sequencing (SLAF-Seq)
Source: Front Plant Sci. 2021 Jun 24;12:688444. doi: 10.3389/fpls.2021.688444 (PMC8264369; doi:10.3389/fpls.2021.688444)
Supplement: Supplementary Appendix — Code availability. [file Data_Sheet_2.PDF]

Main steps of variants (SNPs) calling

bwa: 0.7.10-r789

1. Construction of reference genome index: bwa index -a is ref.fa

2. Comparison with the reference genome using bwa mem-M:
bwa mem ref.fa 1.fq 2.fq -t 6 -M -R "@RG\tID:sample\tLB:R01\tPL:ILLUMINA\tSM:sample"

3. Conversion of sam to bam files using SortSam.jar in Picard tools

Default parameters used in bwa:
-A INT: score for a sequence match, which scales options -TdBOELU unless overridden [1]
-B INT: penalty for a mismatch [4]
-O INT[,INT]: gap open penalties for deletions and insertions [6,6]
-E INT[,INT]: gap extension penalty; a gap of size k cost '{-O} + {-E}*k' [1,1]
-L INT[,INT]: penalty for 5'- and 3'-end clipping [5,5]
-U INT: penalty for an unpaired read pair [17]
-k INT: minimum seed length [19]

GATK v3.8

1. Picard tools: MarkDuplicates.jar

2. GATK tools: Identification of regions for comparison using RealignerTargetCreator and adjusted using IndelRealigner

3. Use HaplotypeCaller to generate gvcf:
java -XX:ParallelGCThreads=5 -Djava.io.tmpdir=tmp -Xmx20G -jar GenomeAnalysisTK.jar -T HaplotypeCaller -R ref.fa --indelSizeToEliminateInRefModel 50 --emitRefConfidence GVCF --variant_index_type LINEAR --variant_index_parameter 128000 --sample_ploidy 4 -nct 4 -o *.g.vcf -L *.chr_allocation.1.list -I *.dedup.realn.bam

4. Use CombineGVCFs to combine GVCF files
java -XX:ParallelGCThreads=5 -Djava.io.tmpdir=tmp -Xmx30G -jar GenomeAnalysisTK.jar -T CombineGVCFs -R ref.fa --disable_auto_index_creation_and_locking_when_reading_rods -o cohort.*.g.vcf --variant *.vcf --variant *.g.vcf --variant *.g.vcf --variant *.g.vcf --variant *.g.vcf

5. Use GenotypeGVCFs to generate variants (SNPs, vcf)
java -XX:ParallelGCThreads=5 -Djava.io.tmpdir=tmp -Xmx50G -jar GenomeAnalysisTK.jar -T GenotypeGVCFs -nt 1 -R ref.fa --disable_auto_index_creation_and_locking_when_reading_rods -o combine.*.snp.indel.vcf

6. Filtering low-quality SNPs:
   (1) bcftools: varFilter -w 5 -W 10

(2) clusterSize 2 clusterWindowSize 5
(3) QUAL 30
(4) QD 2.0
(5) MQ 40
(6) FS 60.0
(7) other default parameters in GATK
